# Cytomegalovirus-induced oncomodulation drives immune escape in glioblastoma

**DOI:** 10.1038/s41598-025-10107-w

**Published:** 2025-07-17

**Authors:** Harald Krenzlin, Felix Corr, Deepak Ailani, Philipp Einheuser, Thomas Bukur, Thomas Rößler, Alina Henrich, Raja Hollnagel, Alice Dauth, Libo Hu, Leon Schmidt, Marion Griessl, Michael Gutknecht, Noe Mercado, Beat Alessandri, Charles H. Cook, Florian Ringel, Sean E. Lawler, Niels A. Lemmermann, Naureen Keric

**Affiliations:** 1 Department of Neurosurgery, University Medical Center Mainz, Mainz, Germany; 2 Department of Neurosurgery, University Medical Center Schleswig-Holstein, Lübeck, Germany; 3 Research Center for Immunotherapy (FZI), University Medical Center, University of Mainz, Mainz, Germany; 4 TRON gGmbH - Translational Oncology at the University Medical Center of the Johannes Gutenberg University, Freiligrathstr. 12, 55131 Mainz, Germany; 5 Department of Surgery, Beth Israel Deaconess Medical Center, Harvard Medical School, Boston, MA USA; 6 Department of Pathology and Laboratory Medicine, Department of Neurosurgery, Legorreta Cancer Center, Brown University, Providence, RI USA; 7 Institute for Virology, University Medical Center Mainz, Mainz, Germany; 8 Institute of Virology, University Hospital Bonn, Bonn, Germany

**Keywords:** Glioblastoma, Cytomegalovirus, CMV mouse model, Immune evasion, MHC I, Immune evasion, Cancer microenvironment, Tumour immunology, Tumour virus infections

## Abstract

Immune evasion and suppression lead to unchecked tumor growth in glioblastoma. Cytomegalovirus (CMV) has been implicated in tumor progression and modulation in glioblastoma. To investigate this potential connection, CMV-associated changes in the glioblastoma immune landscape were characterized in vitro and in a murine glioblastoma model. Infection of mouse glioblastoma cells (GL261Luc2) with mCMV resulted in a short period of viral replication. MHC-I cell surface expression was reduced after mCMV infection by approximately 40% compared with non-infected tumor cells (*p* < 0.0001). Viral regulators of antigen presentation (vRAP) were shown to be responsible for MHC-I downregulation using a recombinant mCMV (ΔvRAP) lacking the known immune evasion genes. RNA sequencing of mCMV infected GL261Luc cells revealed 2711 differentially expressed genes (*p* < 0.005). Of particular interest was the downregulation of MHC-I-associated genes *H2-Q1-10* and *Tap1* fter CMV infection. In vivo, the mCMV immediate early gene (IE1) was detected in brains of mCMV + animals after tumor implantation and increased during tumor growth. mCMV + mice had significantly shorter survival than controls, depending on initial tumor size (*P* < 0.001). Tumor immune infiltrates in mCMV infection were characterized by B cell infiltrates and low levels of NK cell infiltration. Here, the landscape of immune cell infiltrates is shifted toward B cell infiltration and reduced numbers of NK cells. CMV leads to immune evasion mediated MHC-I downregulation in murine glioblastoma. Thus, CMV infection in glioblastoma may contribute to unchecked tumor growth in glioblastoma by increasing immune evasion.

**Supplementary Information:**

The online version contains supplementary material available at 10.1038/s41598-025-10107-w.

## Introduction

Glioblastoma is the most common malignant primary tumor of the central nervous system (CNS). It is a devastating and universally fatal disease with a dismal median overall survival of only 15 months^1,2^. Standard treatment consists of surgical excision, radiation, and alkylating chemotherapy with temozolomide (TMZ)^3^. Therapeutic resistance and tumor recurrence after resection contribute to poor prognosis^4^, and intratumoral heterogeneity and tumor cell plasticity add to the complexity of tumor treatment^5^.

Traditionally, the brain has been considered immune-privileged in part due to the existence of the blood-brain barrier^6–9^. Microglia, the resident innate immune cells of the CNS, do not express major histocompatibility complex class II (MHC-II) in the resting state, but can be activated in certain scenarios such as autoimmune encephalitis to become MHC-II expressing antigen-presenting cells (APCs)^10^. Furthermore, microglia and macrophages cross-present antigens via MHC class I to CD8 + T cells, and local CD11c + dendritic cells are crucial for CD4 + T cell infiltration^11^. Despite these and other known interactions between the brain and the immune system, the CNS remains immunologically unique^9^.

Glioblastoma-mediated immunosuppression is severe involving the tumor-microenvironment (TME) and even extends to peripheral immune organs^12,13^. Engagement of immune checkpoint pathways by the tumor is one of the major mechanisms by which immune resistance to tumor antigen-specific T cells develops^14^. Invading glioma cells are also thought to escape immune recognition primarily via MHC-II downregulation^15^. Within the tumor TME, expression of immunosuppressive cytokines such as IL-6, IL-8, IL-10 and TGF-ß contribute to MHC downregulation^13,16,17^.

Human cytomegalovirus (HCMV) is a member of the β-herpesvirus family which is widespread in human populations with a prevalence of 60–90%. Following acute infection, HCMV persists lifelong in a dormant state referred as latency. During this, productive infection is suppressed but viral genomes remain at specific cellular sites where replication can be reactivated^18^.

HCMV was first reported to be present in glioblastoma patient tumor specimens in 2002^19^. In subsequent studies, various HCMV genes have been implicated in tumor development and progression^20–23^. There is evidence, that HCMV seropositivity in patients with glioblastoma is associated with poorer overall survival (OS)^24^. A role for CMV in tumor promotion has been supported by studies in mouse models that show accelerated tumor growth in the context of latent CMV infection, which is associated with local CMV reactivation, and a pro-angiogenic/mesenchymal phenotype, that can be reversed with anti-viral therapy^23^. Increasing evidence supports a direct oncogenic role for HCMV in glioblastoma. HCMV gene products can drive malignant phenotypes; for example, the viral IE1 protein enhances glioma stemness and EMT-associated transcription factors in infected glioblastoma cells^25^. Recent studies showed that human astrocytes exposed to HCMV acquire tumorigenic properties and, importantly, can generate glioblastoma-like tumors upon xenotransplantation in animal models^26,27^. These studies support HCMV as not merely an oncomodulator but a direct contributor to glioblastoma oncogenesis.

The effects of CMV on immunosuppression in glioblastoma have not been well studied. The aim of this study was to analyze mCMV-induced changes to mechanisms of immune suppression and evasion in a mouse glioblastoma model. We hypothesized that the immunosuppressive TME associated with glioblastoma provides a supportive environment for CMV reactivation^28^. Immunosuppression could be further compounded because the CMV genome encodes numerous immunoevasive proteins and microRNAs to allow virus replication despite competent innate and adaptive arms of the immune system^29^. Tumor recognition by CD8 + T cells requires antigen presentation on MHC-I molecules and downregulation of cell surface MHC-I molecules is considered as a hallmark function of CMV-encoded immunoevasins^29^. Immunoevasins are a set of glycoproteins whose only known function is to prevent the presentation of antigenic peptides by the MHC class I^30^. Our data support this concept suggesting that CMV reduces expression of MHC-I molecules on glioblastoma cells blocking anti-tumor immunity, making them resistant to T cell-mediated killing and thus contributing towards tumorigenesis, progression and resistance to therapy.

## Materials and methods

### Cell lines and viruses

GL261Luc2 murine GBM cells were purchased from Perkin-Elmer (Boston, MA) and cultivated in in Dulbecco’s Modified Eagle’s Medium (DMEM, D6046; Sigma; USA), supplemented with 10% fetal calf serum (Biological Industries, Israel), and 1% penicillin-streptomycin (P4333; Sigma; USA) at 37 °C in a humidified incubator with 95% air humidity and 5% CO_2_. NIH3T3 mouse fibroblasts used for virus propagation, were purchased from ATCC and cultured in DMEM with 10% FBS (Sigma-Aldrich), penicillin (100 U/ml), and streptomycin (10 mg/ml) (Thermo Fisher). Mycoplasma testing was routinely performed by PCR, and STR profiling was used to confirm cell identity.

mCMV (Smith strain, ATCC VR-1399, American Type Culture Collection, Manassas, VA, USA) high titer virus stock was purified from cell culture and virus titers were determined as previously described^23^. High titer virus stocks of BAC-derived mCMV-wt^31^, mCMV-Δm157eGFP^32^, and mCMV-Δm04m06m152^33^ were generated as described previously^34^. Infection of newly born (P2) mouse pups was carried out as previously described^23^.

### Growth in low attachment (GILA) and cell cycle analysis

The cellular density for the assay was optimized at 1,000 GL261Luc2 cells per well in 100 µL of medium. Equal numbers of cells were seeded into wells of an ultralow-attachment 96-well plates, grown for 9 d, and analyzed using light microscopy. For cell cycle analysis, 5 × 10^5^ cells suspended in 0.25 mL PBS and 200 µL of Muse Cell Cycle Reagent (Merck, Millipore, Billerica, MA, USA), were incubated for 30 min at room temperature, protected from the light. Cell cycle was assayed using a Muse Cell Analyzer (Merck, Millipore, Billerica, MA, USA).

### CMV infection in vitro

For in vitro infection, we seeded up to 10^6^ GL261Luc2 cells in six-well plates and treated the cells with CMV or mock (purified extract from uninfected fibroblasts) the next day. Cells were infected with either mCMV-Δm157eGFP, mCMV-wt, or mCMV-ΔvRAP with different multiplicities of infection (MOI) as described previously^23,33,35^. For UV irradiation, virus stock was placed in 60 mm plastic Petri dishes and exposed to a 15 W short-wavelength UV light (UV crosslinker, Boekel Scientific, Feasterville, PA, USA). Inactivation was achieved by delivering an energy dose equivalent to 100.000 µJ.

### Plaque assay

Monolayers plated in 12-well plates the previous day were infected with mCMV-Δm157eGFP at different MOIs. Virus was adsorbed for 3 h. Culture medium was then removed and covered with 1.5% carboxymethyl cellulose (Sigma) and DMEM supplemented with 10% FCS (1:1). Cells were incubated for up to 7 days to allow plaque formation.

### mCMV genome quantitation

To determine viral genomes, DNA extracted with the DNeasy blood and tissue kit (catalog no. 69504; Qiagen, Hilden, Germany) according to the manufacturer’s instructions. Viral and cellular genomes were quantitated in absolute numbers by M55-specific and pthrp-specific qPCRs normalized to a log_10_-titration of standard plasmid pDrive_gB_PTHrP_Tdy^36^.

### Quantitative RT-PCR

Quantitative RT-PCR (qRT-PCR) was performed as previously described^23^. Total RNA was extracted using Trizol (Life Technologies, Carlsbad, CA, USA) and treated with RNase-free DNase (Qiagen, Germany). mRNA expression analysis was carried out using Power SYBR Green (Applied Biosystems, CA). RNA concentration was quantified using a Nanodrop RNA 6000 (ThermoFisher) and analyzed using the STEP one PLUS Applied Biosystems PCR machine. Primer sequences were as follows.

TGF-ß: FOR 5’- GACCGCAACAACGCCATCTA − 3’, REV 5’- GGCGTATCAGTGGGGGTCAG − 3’; IL-6: FOR 5’- CCGGAGAGGAGACTTCACAG − 3’; REV 5’- GGAAATTGGGGTAGGAAGGA − 3’; IL-8: FOR 5’- GCTGGATCACACTGCAGAAA − 3’, REV 5’- TCAAGGAAAAGTTTGCAGCA − 3’; VEGF-A: FOR 5’-AACGATGAAGCCCTGGAGTG − 3’, REV 5’- GACAAACAAATGCTTTCTCCG − 3’; HGF: FOR 5’- TTAAAACGTGCGCTCACAGTG − 3’, REV 5’- GGTATTGCTGGTTCCCCTGTAA − 3’; PDGF-A: FOR 5’- GTCCAGGTGAGGTTAGAGG − 3’, REV 5’- CACGGAGGAGAACAAAGAC − 3’; PDGF-B: FOR 5’- TGAAATGCTGAGCGACCAC − 3’, REV 5’- AGCTTTCCAACTCGACTCC − 3’; PDGF-C: FOR 5’- AGGTTGTCTCCTGGTCAAGC − 3’, REV 5’- CCTGCGTTTCCTCTACACAC − 3’; PDGF-D: FOR 5’- CCAAGGAACCTGCTTCTGAC − 3’, REV 5’- CTTGGAGGGATCTCCTTGTG − 3’; IE1: FOR 5’- AGCCACCAACATTGACCACGCAC − 3’, REV 5’- GCCCCAACCAGGACACACAACTC − 3’; gB: FOR 5’- ATCTCGTCCAGGCTGAACAC − 3’, REV 5’- TCATCAACTCGACGAAGCTC − 3’.

### Mouse GBM models

Mouse GBM models were established as previously described^23^. Briefly, pups of C57BL/6 mice were infected i.p. with 10^3^ pfu mCMV at day 2 post natum. After 14 weeks 1000 GL261Luc2 tumor cells were implanted intracranially .

### Flow cytometry

Anti-MHC class I (Biorad; MCA2189A647) and anti-MHC class II (Biorad; MCA2401A488) monoclonal antibodies were used at 1:25 dilution. Cells were harvested and washed before staining with appropriate fluorophore-conjugated antibodies for flow cytometry. For peptide staining, a final concentration of 5 µM was used. Staining was performed at 4 °C for 30 min. Cells were then washed and fixed in 2% paraformaldehyde. Acquisition of data was performed on an Attune Acoustic Focusing Cytometer (Applied Biosystems, Waltham, MA, USA) and FCS Express 7 software version 10.0.5 (TreeStar, San Carlos, CA, USA) was used to analyze the mean fluorescent intensity (MFI) and cell percentages. The MFI represents the median fluorescence as calculated by the software. For each staining condition, the respective MFI of unstained/isotype control was subtracted.

### Immunofluorescence

GL261Luc2 cells were seeded onto glass coverslips precoated with poly-d-lysine (Thermo Fisher Scientific) in 6-well plates and allowed to adhere overnight. After reaching approximately 70% confluence, cells were infected with the indicated virus. Cells were fixed with 4% paraformaldehyde for 15 min at room temperature and then washed with PBS. Cells were then permeabilized with 0.2% Triton X-100 in PBS for 10 min and then blocked with 5% BSA in PBS for 1 h at room temperature to reduce nonspecific binding. Primary antibodies against MHC I, MHC II, CMV (Virusys Corporation; CA150-1), and pp65 (ABIN727070) were used at a dilution of 1:100 and incubated overnight at 4 °C. After washing with PBS, cells were treated with Alexa-Fluor-conjugated secondary antibodies for 1 h at room temperature. Cell nuclei were counterstained with DAPI (Thermo Fisher Scientific) for 5 min. Coverslips were mounted on glass slides with embedding medium and sealed. Images were acquired using a fluorescence microscope (Leica Thunder Widefield Fluorescence Microscope) with appropriate filter sets. Multiple fields of view were acquired to obtain representative images. Negative controls without primary antibodies were used to assess background fluorescence.

### RNA sequencing

Cell sorting was conducted at the Flow Cytometry Core Facility, IMB Institute of Molecular Biology, Mainz, Germany. GL261Luc2 cells were cultured at a density of 1 × 10^6^ cells in six-well plates, infected with mCMV-Δm157eGFP, dissociated with Accutase, washed, and resuspended in FACS buffer containing 0.2% BSA. Utilizing a flow cytometer equipped with a 488 nm laser for GFP excitation and emission detection via a 530/30 nm bandpass filter, GFP + GL261Luc2 cells were discriminated based on distinctive forward scatter and GFP fluorescence profiles. A sorting threshold was established specifically for GFP + cells, enabling the selective sorting of exclusively mCMV-Δm157-infected cells. All samples were acquired on the BD FACS Aria Fusion III (BD Biosciences) and analyzed using the BD FACSDiva 8.0.2 software and FlowJo(v10, FlowJo LLC). Sequencing of GL261Luc2 before and after infection with mCMV was performed as previously described^23^. All RNAseq data have been deposited in the European Nucleotide Archive (ENA) at EMBL-EBI under accession number PRJEB85259 (https://www.ebi.ac.uk/ena/browser/view/PRJEB85259).

### Statistical analysis

Imaging assays were quantified using ImageJ (http://rsb.info.nih.gov.ezpprod1.hul.harvard.edu/ij/), including the Analyze Particles function of binary images with an automatic threshold. Data are expressed as mean ± SD. Unpaired two-tailed Student’s t-test was used for comparison between two groups. Each group was tested for a Gaussian distribution, if one-way ANOVA was passed, followed by Bonferroni’s test. If this failed, the Kruskal-Wallis test followed by Dunn’s correction was conducted to test for significance of differences among multiple groups. Pearson’s correlation with nonlinear regression analysis was performed to compute Pearson’s r and p-values. Statistical analyses were performed using Microsoft Office Excel 2011 or GraphPad Prism 6 software. *P* < 0.05 was considered statistically significant.

A t-distributed stochastic neighbor embedding (tSNE) plot was created of immune infiltrates in tumors from mCMV + mice to evaluate the immune landscape.

## Results

### mCMV replicates to a limited extent in murine glioblastoma cells

**Fig. 1:**
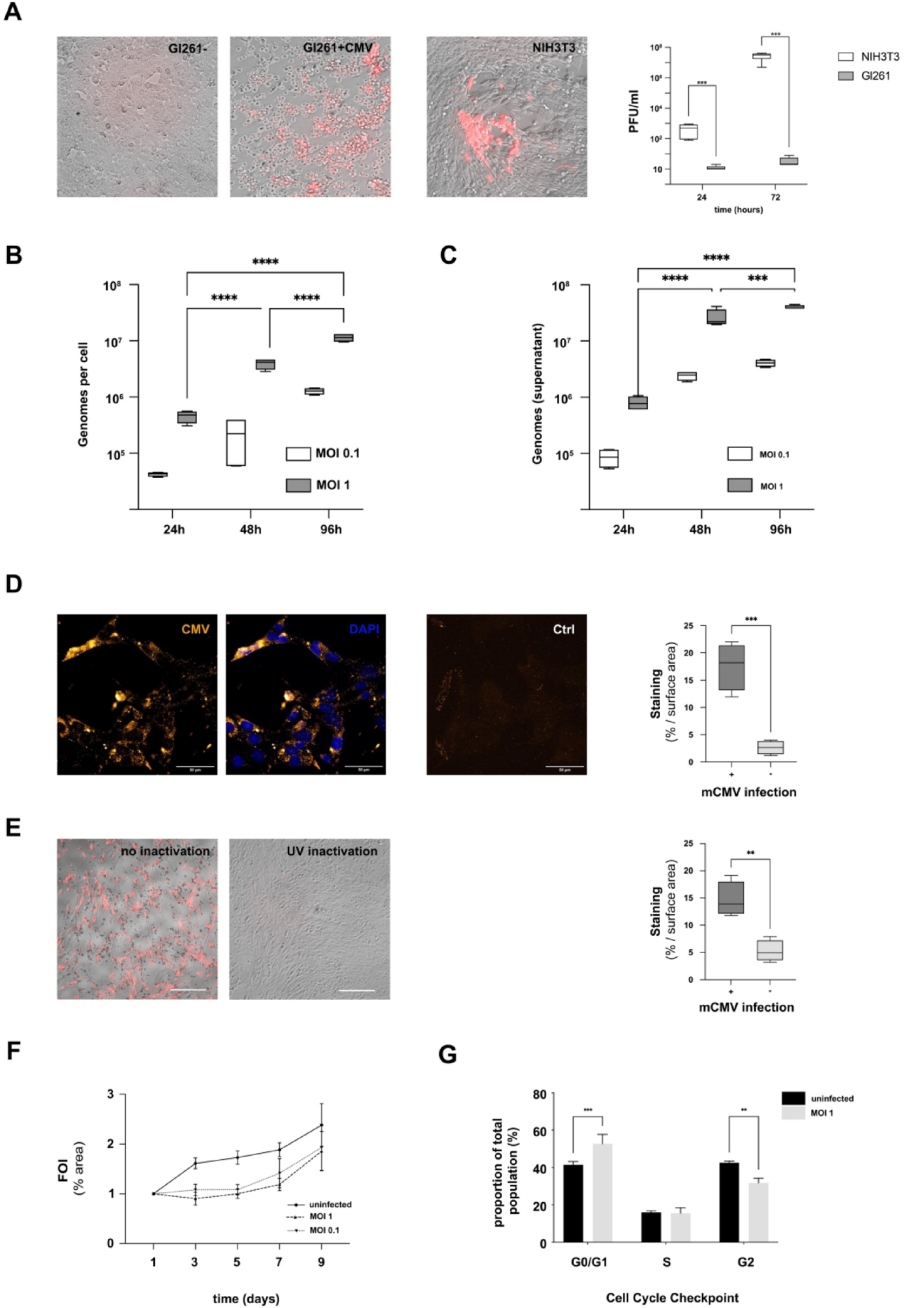
Characterization of CMV induced changes to murine glioblastoma cells in vitro. (**A** +  **E**) GL261Luc2 mouse glioblastoma cells were infected using mCMV-Δm157mCherry. Red florescence indicated viral infection. No plaque formation was detected in GL261Luc2. (middle) NIH3T3 are shown as positive control. (right). No fluorescence was detected after previous irradiation of virus stocks using UV light. (*p* = 0.0024) (**B**) Viral copy number increase intracellular and (**C**) in supernatant (*p* = 0.0001). (**D**) mCMV-associated pp65 homolog antigen was detected using immune staining (**E**) Proliferation measurement of 3D cell cultures in ultra-low attachment plates. (**F**) The distribution of cells among the cycle phases 72 h after infection with mCMV at MOI 1 (S phase: *p* = 0.9973; G_0_/G_1_, G_2_: *p* = 0.0009 and *p* = 0.0013).

The mechanisms behind the establishment, persistence, and dissemination of CMV infection in glioblastoma remain poorly understood^37^. Our previous data showed accelerated tumor growth using the mouse GL261 GBM model after CMV infection in vivo. To understand this process in more detail, GL261Luc mouse glioblastoma cells were infected with mCMVΔ157eGFP and mCMVΔ157mCherry in vitro. The mCherry labeled virus was utilized to detect lytic replication by plaque formation. Only very low numbers of plaques were detected compared to NIH3T3 fibroblasts as controls. This finding indicates that GL261 murine glioblastoma cells do not support efficient lytic replication after mCMV infection. (Fig. 1A) To detect signs of viral replication, tumor cells and supernatants were harvested at different time points after mCMV infection. After infection with MOI 1, intracellular viral copy numbers increased from 24 h (8.02 × 10^5^ ±2.3 × 10^5^) to 48 h (2.62 × 10^7^ ±1.0 × 10^7^, *p* < 0.0001) and 96 h (3.99 × 10^7^ ±3.4 × 10^6^, *p* < 0.0001). The increase in viral copy number was not statistically significant at a lower MOI of 0.1 at 24 h (8.5 × 10^3^ ±3.3 × 10^4^), 48 h (2.4 × 10^6^ ±4.5 × 10^5^, *p* = 0.9753) and 96 h (4.04 × 10^6^ ±5.9 × 10^5^, *p* = 0.9959). With both MOIs, replication was highest within the first 48 h and markedly declined thereafter. Similar patterns, although less pronounced, were found during analysis of viral copy numbers in the supernatant. Viral copy numbers in the supernatant increased from 24 h (4.5 × 10^4^) to 48 h (3.9 × 10^5^, *p* = 0.0002) and 96 h (1.1 × 10^6^, *p* < 0.0001) after infection with MOI 1. The increase in viral copy number was not statistically significant at a lower MOI of 0.1 at 24 h (4.2 × 10^3^ ±384.2) to 48 h (2.2 × 10^4^ ±1.9 × 10^4^, *p* < 0.3312) and 96 h (1.2 × 10^5^ ±1.6 × 10^4^, *p* < 0.5076). (Fig. 1B, C) Using immunofluorescence, CMV-associated antigens were detected after infection. (Fig. 1D). No infection was detected after previous irradiation of virus stocks using UV light. (Fig. 1E). Proliferation was found to be decreased immediately after infection (MOI 1 *p* = 0.0006; MOI 0.1 *p* = 0.0001) and recovered 3 days after infection. (Fig. 1F) The distribution of cells among the cycle phases G_0_/G_1_, S, and G_2_ 3 days after infection with mCMV at MOI 1 was similar to uninfected cells. (Fig. 1G).

### Transcriptomics reveals alterations in immune related genes after mCMV infection of GL261Luc2 cells

**Fig. 2:**
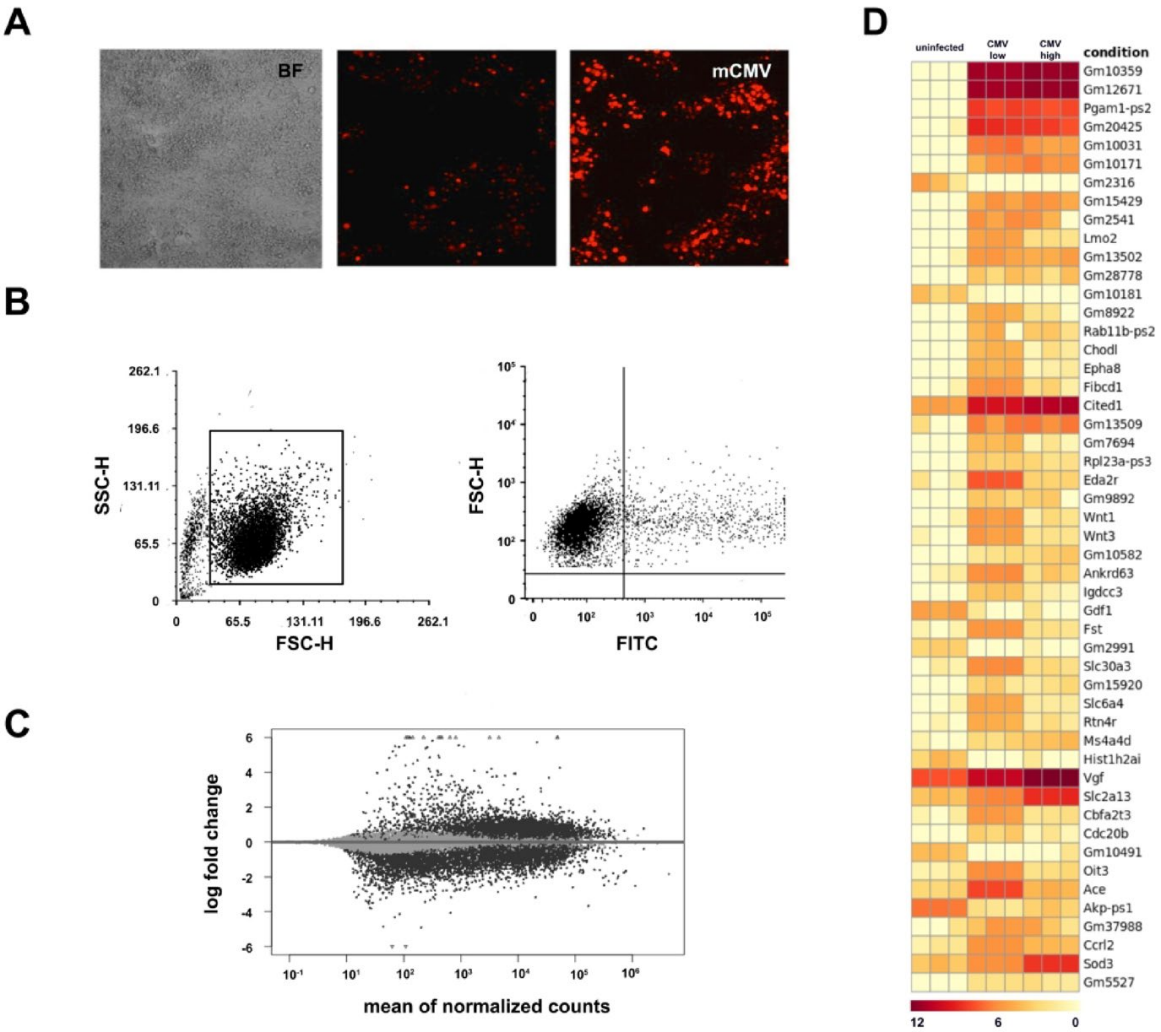
Transcriptome changes in mouse glioblastoma cells (Gl261) after mCMV infection. (**A**) Gl261 cell infected with mCMV-GFP (left: bright field (BF); middle: 24 h; right: 48 h after infection) (**B**) GFP-positive cells and GFP-negative cells were sorted using a fluorescence-activated cell sorting (FACS) system. (**C**,**D**) RNA-seq of mCMV infection in murine glioblastoma cells 48 h post-infection. A total of 2711 genes were differentially expressed with 316 more than two-fold (log2) after mCMV infection of GL261Luc murine glioblastoma cells (p adjusted < 0.005).

To visualize CMV infected GL261Luc2 cells, mCMVΔm157eGFP was used to allow imaging of infection via fluorescence microscopy. After infection of cells at 80% confluency with MOI 1, only a fraction of cells were infected as indicated by GFP positivity. After 24 h, approximately 5% of cells were GFP- positive, while after 48 h post-infection half of all cells showed GFP fluorescence. (Fig. 2A). GFP-positive cells and GFP-negative cells were sorted by FACS and RNA-seq was performed on each sample 48 h post-infection. The analysis was performed on GFP-positive and negative cells separately. (Fig. 2B) A total of 2,711 genes were differentially expressed with 316 more than two-fold (log_2_) after mCMV infection of GL261Luc murine glioblastoma cells (p adjusted < 0.005) (*n* = 3) (Fig. 2C). Comparison of altered genes from infected cells (GFP low and high) with uninfected cells revealed similar altered genes set independent from GFP reporter expression (Fig. 2D). Increased secretion of cytokines such as IL-6, IL-8, and TGF-β was observed in response to CMV infection (Supplementary Fig. 1).

Of particular interest was the downregulation of MHC-I-associated genes H2-Q1-10 and Tap1 after CMV infection (8.13 log2, *p* < 0.005, not shown). Because of its known importance in glioblastoma immune evasion, we investigated the functional consequences of mCMV-induced MHC regulation in more detail.

### Downregulation of MHC I/II after mCMV infection

**Fig. 3:**
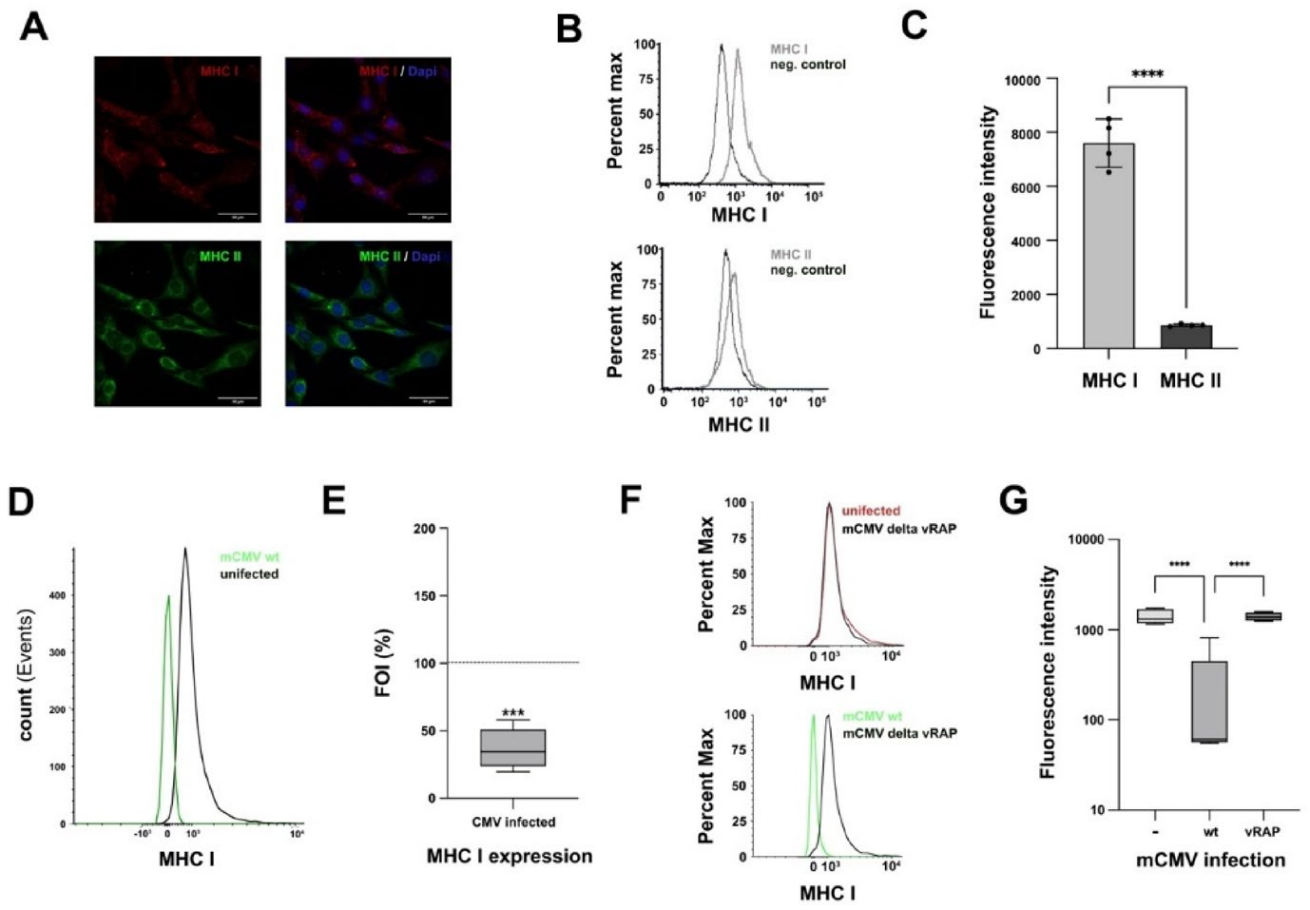
MHC-I/II expression in mouse glioblastoma cells. (**A** + **B**) The expression of MHC I (red) and MHC II (green) in mouse glioblastoma cells (*p* < 0.0001) (**B**,**C**). Cell surface expression of MHC class I and MHC class II detection using flow cytometry. (**D**,**E**) Downregulation of MHC class I after infection with mCMV wt (**F**,**G**). Immunevasin deletion mutant mCMV (mCMV—ΔvRAP) was used to analyze vRAP dependent MHC I downregulation.

To determine the ability of mouse glioblastoma cells to present viral and tumor antigenic peptides, expression levels of MHC-I/II on the cell surface were assessed using immunostaining and flow cytometry in two different glioblastoma cell lines. Cell surface staining of MHC class I molecules was stronger than that of MHC class II as determined by fluorescence microscopy (Fig. 3A). Flowcytometry was used to determine to determine difference in baseline MHC expression levels. The expression of MHC class I molecules was approximately 8-fold higher than that of MHC II (*p* < 0.0001) in uninfected cells compared to negatve controls (Fig. 3B and **C**). In mCMV infected cells, immunevasine expression occurs during E-phase up to 16 h after infection^38^. 24 h after infection with mCMV-wt, expression of MHC class I was reduced to approximately% compared with uninfected cells (*p* < 0.0001) (Fig. 3D, E).

### CMV induced MHC downregulation through immunoevasin in glioblastoma cells

MHC-I downregulation after mCMV infection is mediated through “viral regulators of antigen presentation” (vRAP)^30^. Immunoevasin deletion mutant mCMV (mCMV-ΔvRAP) was used to analyse vRAP dependent MHC cell surface expression. Following infection of GL261Luc2 with mCMV-ΔvRAP no downregulation of MHC-I was detected compared to mCMV-wt virus (Fig. 3F). Despite mCMV-ΔvRAP infection tumor cells retained MHC-I expression levels similar to uninfected cells. (Fig. 3G). This might indicate that viral imunoevasins can play a role in regulation of MHC-1 expression in glioma cells.

### Effects of mCMV infection in a mouse glioblastoma model

**Fig. 4:**
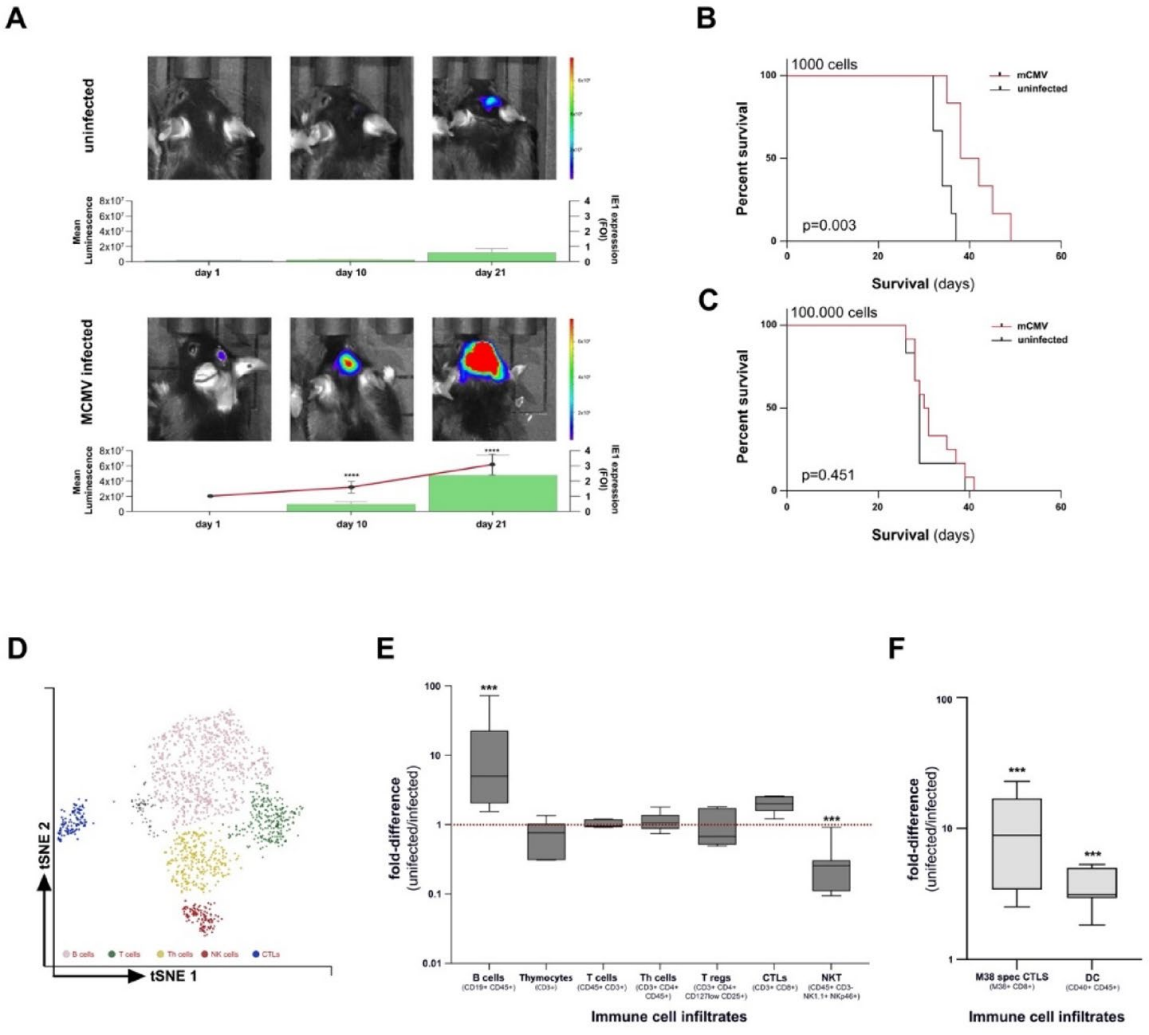
Tumor growth and immune infiltrates in vivo. C57BL/6 mice were infected 2 days after birth (P2) with mCMV (mCMV+). Orthotopic tumors (luciferase-expressing GL261Luc2) were stereotactically implanted at least 15 weeks after infection. The expression of immediate early gene (IE-1) was detected in the brain of mCMV + animals, but not in mCMV naïve. Transcripts of both the immediate early IE-1 and the envelope-associated glycoprotein B (gB) (not shown) were not detected prior to tumor cell injection, then increased during tumor growth. Bioluminescence imaging (BLI) and T2-weighted magnetic resonance imaging (MRI) revealed significantly faster tumor growth in mCMV + mice than in controls (**A**). Larger tumor volumes lead to earlier weight loss and shorter survival in mCMV + mice. (*p* < 0.001) (**B**) Effect of mCMV dependent on initial tumor mass with mechanisms of immune evasion might become predominant over the rapid proliferation and consecutive mass effect in primarily larger tumors (**C**). t-distributed stochastic neighbor embedding (tSNE) plot of immune infiltrates in tumors from mCMV + mice. (**D**) Tumors of mCMV + mice are characterized by higher numbers of B cell infiltrates (*p* = 0.0197) and lower levels of NK cell infiltration (*p* = 0.0027) compared to uninfected controls (**E**). mCMV leads to the infiltration of mCMV specific CTLs and dendritic cells (*p* = 0.0196) (**F**).

To investigate whether mCMV can spread in vivo to brain tumor cells implanted into uninfected hosts, C57BL/6 mice were infected 2 days after birth (P2) with mCMV-Δm157 (mCMV+). Orthotopic tumors (lGL261Luc2) were stereotactically implanted at 14 weeks after infection, a time when viral latency is known to be established. To detect early reactivation viral transcriptional immediate early 1 (IE1) expression has been measured after tumor implantation by RT-qPCR. IE1 induction was detected in the brain of mCMV + animals, but not in mCMV naïve ones. Ten days after tumor implantation, IE1 expression was doubled (*p* < 0.0061) and increased up to 3-fold at day 21 (*p* < 0.0002) in comparison to mCMV + animals without tumor implantation (Fig. 4A). Transcripts of both the immediate early IE1 and the envelope-associated glycoprotein B (gB) increased in brains of mCMV + mice during tumor growth, suggesting mCMV reactivation after tumor implantation. Bioluminescence imaging (BLI) and T2-weighted magnetic resonance imaging (MRI) revealed significantly faster tumor growth in mCMV + mice than in controls (Fig. 4A). Larger tumor volumes lead to earlier weight loss and clinical deterioration. mCMV + mice had a significantly shorter survival than controls, which depended on the initial tumor cell load (*p* < 0.001) (Fig. 4**BC**). Thus, our data show that the presence of preexisting mCMV infection is associated with more rapid tumor mass-dependent tumor growth in a mouse model.

### B cells dominate the immune landscape of mCMV + GL261 tumors

Immune infiltrates were evaluated in brain tissues in tumor bearing hemispheres relative to contralateral normal brain and compared between mCMV infected and mCMV naïve animals. A t-distributed stochastic neighbor embedding (tSNE) plot was created of immune infiltrates in tumors from mCMV + mice. Phenotypically similar cells were clustered in an unsupervised manner. (Fig. 4D) Tumors of mCMV + mice are characterized by B cell infiltrates and low levels of NK cell infiltration (Fig. 4E). However, mCMV leads to the infiltration of mCMV specific CTLs and dendritic cells (Fig. 4F)^39^. These data support an immunomodulatory role of CMV infection which can be observed both in vitro and *in vivo.*

## Discussion

HCMV has long been associated with glioblastoma and linked with a poor prognosis^6,24^. However, the underlying biological associations and mechanisms remain largely unknown. The presented data indicates a possible link between mCMV and MHC downregulation in glioblastoma. CMV is notoriously hard to detect in glioblastoma samples^40^. In this study, infection of mouse glioblastoma cells supports the idea of short-term viral replication in these cells. These experiments provide evidence that tumor establishment in a mCMV infected host leads to viral reactivation, increase of viral antigens and accelerated tumor growth in a murine orthotopic glioblastoma model in vivo. The transcriptomic changes observed in this model were similar to those in human glioblastoma cells^22,23^. We showed that downregulation of MHC class I and II in infected tumor cells can be mediated by the vRAPs of mCMV. These changes suggest that CMV might contribute to tumor immune evasion in glioblastoma.

### CMV leads to MHC downregulation in glioblastoma cells

Immune evasion is a hallmark of both, glioblastoma and CMV. Given that immune evasive mechanisms of CMV and glioblastoma are quite similar, it is speculated that they might reinforce each other. The presented data demonstrated MHC class I and II surface expression on murine glioblastoma cell lines, rendering them capable of antigen presentation. Although constitutively low, further MHC class I and II downregulation and secretion of transforming growth factor (TGF)-β were observed after CMV infection in vitro. This decrease in antigen presentation capacity, together with suppression of the adaptive and the innate immune systems by TGF-β could lead to enhanced immune evasion not only against viral but also tumor antigens in the wake of glioblastoma CMV infection.

Previous reports suggested that there is MHC class I expression, but not class II, in GL261 mouse glioblastoma cells^41^. MHC class I and II expression, although present in glioblastoma, is lower than in non-CNS tissue in vitro and in vivo^7,9,42^. This contributes to tumor immune evasion through bypassing immune recognition^42^. MHC class I and II downregulation occurs during all phases of HCMV infection^43,44^. To avoid cell killing by the lack of MHC-I-mediated licensing and failure of self-recognition, altered-self MHC class I molecules are partially rescued^45,46^. This might explain why MHC class I is significantly reduced but not abolished in mouse glioblastoma cells after mCMV infection. T cell activation depends on co-stimulation of either CD4 or CD8 together with T cell receptor (TCR) antigen recognition^47^. Evidence suggests that MHC II-restricted antigen presentation is a pivotal mechanism to directly maintain functional cytotoxic T cell states in brain tumors^48^. It has been shown that MHC class II is essential for the activation of CD4 + T cells and its deficiency might contribute to an exhausted phenotype of tumor-reactive CD8 + T cells^48,49^. The downregulation of MHC class I and secretion of TGF-β are known to suppress the cytotoxic anti-tumoral response and T-cell proliferation^50^. While loss of MHC class I expression is a strong signal of NK cell recognition, soluble factors such as TGF-β secreted by tumor cells, impair NK cell function. The presented data suggest concerted changes at the cellular level and in the TME that allow evasion of the immune system and consecutive tumor growth.

### MHC downregulation is driven by viral regulators of antigen presentation

Expression of CMV immunoevasins leads to limited antigen presentation to T cells and facilitates viral infection^30,51^. However, unlimited viral spread and cytopathogenic infection is prevented by a competent immune system^18,52^. In times of immune compromised conditions such as immunosuppressive graft-vs.-host disease (GvHD) prophylaxis, whole brain radiation or within the glioblastoma microenvironment, reactivation of latent infection can occur^46,53^. Here, glioblastomas in CMV seropositive patients express less surface bound MHC-I. This downregulation can be induced through mCMV infection of mouse glioblastoma cells in vitro. In MCMV immunevasins are expressed during E-phase with some binding ermantantly to MHC class-I and rerouting it to degradation. After infection, IE-to-E phase transition occurs 30 min, E-to-L phase transition 14–16 h^38^. Using a mutant mCMV with impaired vRAPs prevents MHC-I downregulation. Similar MHC class-I downregulation is expected in dendritic cells and pericytes as well- both reported niche of CMV infection in glioblastoma^23^. These findings suggest a functional association of viral regulation of antigen presentation and immune evasion in glioblastoma. Our data indicates that the initial IE and E phase are take place in glioblastoma without ultimate progression to virion release in the tumor cells- which is not necessarily true for other cells of the tumor micro milieu. The observed IE1 signals may reflect latent or persistent CMV within glioblastoma cells or tumor-associated macrophages. Additionally, it has been hypothesized that CMV could integrate into the host genome at low frequencies, enabling constitutive IE1 expression independent of productive replication^54^. These mechanisms could explain IE1 expression independent of extensive lytic replication. It might be thus that the reduced expression of MHC class I after CMV infection might be linked to glioblastoma immune evasion, more aggressive tumor growth and impaired OS.

Further, TAMs in glioblastoma predominantly exhibit an immunosuppressive phenotype, secreting factors such as IL-10 and TGF-β, thereby supporting immune evasion and tumor growth. Notably, CMV frequently infects monocyte/macrophage lineages and may promote an immunosuppressive M2 phenotype through viral cytokines such as cmvIL-10^54^. Consequently, CMV-infected TAMs likely intensify the immunosuppressive microenvironment in glioblastoma.

### Mouse glioblastoma cells are permissive for mCMV and support to short periods of replication

Detection of CMV in glioblastoma remains challenging, and viral reactivation and spread controversial^37,40^. In vitro studies revealed that neurons and glial cells exhibit strong reporter gene expression after HCMV exposure^55^. In addition, endothelial cells, tanycytes, radial glia, ependymal cells, microglia, cells from the meninges and choroid can be infected by CMV^22,23,55,56^. Brain cultures selectively enriched in either glia or neurons supported viral replication, resulting in process degeneration and cell death within 2 d of viral exposure^55,56^. In the presented data, an increasing amount of viral DNA was detected in infected mouse glioblastoma cell lines and supernatant, suggesting replication in vitro. The increase of viral DNA within the cell subsided 96 h after infection. During this initial period cell growth was halted and recovered thereafter. Lytic replication was not observed in either monolayer or neurosphere cultures. CMV replication likely continues beyond the initial infection period, entering latency and possibly reactivating subsequently. Studies have demonstrated that HCMV strains can alternate between latency and reactivation cycles over prolonged culture periods lasting several weeks^54^. Thus, in our glioblastoma model, viral persistence at low levels and intermittent reactivation is highly plausible. As viral DNA persists within the cell, it is postulated that oncomodulatory effects prevail over the initial detrimental effects of viral infection^22^. In support of our data, similar observations of infected human GSCs not supporting lytic replication have been previously reported^22,23^. Nevertheless, others report all phases of productive (lytic) phase viral gene expression in primary glioblastoma cell lines in vitro for up to 72 h^57^. During this time, the cells divide less frequently and begin to grow again upon further passage. Thus, the reported kinetics are similar to the observation made in this study. So far, no plaque formation as indicator of lytic replication has been reported in glioblastoma cell lines. Once infected, CMV induces phenotypic plasticity and promotes mesenchymal and stem-like features potentially increasing tumor aggressiveness^58^. Primary human glioblastoma cell lines infected in vitro show increased sphere formation capability, indicating a change towards stem-like cell characteristics^57^. This transition is consistent with the expression of mesenchymal markers such as c-MET in GSCs of the proneural subtype^22^. In contrast, brain endothelial cells were found to be permissive to CMV infection and capable of plaque formation in rats^59^. Both pericytes and endothelial cells are believed to support lytic replication and thus facilitate viral spread through the immunosuppressive TME^60,61^. As GSCs give raise to tumor-associated pericytes to support vessel function and tumor growth, the intertwined nature of CMV infection, phenotypic stem cell transformation, and localization towards the perivascular niche is further supported^23,55^. These results indicate oncomodulatory changes of tumor cells toward a more aggressive mesenchymal subtype due to infection with CMV without of lytic replication. This may explain some aspects of the poor clinical outcome observed in CMV-positive patients with glioblastoma.

### CMV leads to increased tumor growth and tumoritropic viral spread

In this study, expression of the viral genes IE1 and gB was detected in orthotopically established tumors using murine glioblastoma cell lines. Both genes have been previously described as suitable reporter for CMV infection^55^. CMV antigens have been identified in tumor specimens using various techniques such as immunostaining, in situ hybridization and polymerase chain reaction^62^. The presented data demonstrate for the first time the in vivo presence of mCMV transcription in naïve tumor cells and their longitudinal course of expression in infected animals after orthotopic tumor implantation. As the prevalence of HCMV infected tumor cells is low, the importance of optimized methods was emphasized by Cobbs et al.^40^ However, detection of viral DNA in tumor samples using PCR or NGS has been diffcult^40,62,63^. These shortcomings may be due to a lack of sensitivity in detecting small amounts of DNA by NGS and the presence of viral DNA in only a minority of glioblastoma cells^64^. One possible explanation for the difficulty in viral DNA isolation is the short period of viral replication in glioblastoma cells without a substantial genome replication and production viral progeny, leading to limited infection of tumor. IE1 is mainly detected within, but not restricted to tumor tissue. In the CNS, IE1 can be mainly localized in the meninges, the choroid plexus (intraventricular infection), within endothelial cells, and pericytes (intravascular infection)^65^. However, the expression of mCMV IE1 and gB within mouse glioblastoma increased continuously during tumor development, providing further proof of the concept of viral spread through cells of the TME, which consists of vascular- and endothelial cells, infiltrating and resident immune cells, and other nonneoplastic glial cells^23,66^. Analysis of the immune cell composition revealed increased numbers of infiltrating B cells and fewer NK cells in mCMV-infected mice compared with the levels in uninfected control mice. It is speculated that the lower number of NK cells infiltrating the tumor may be due to mCMV induced changes in the TME. T cells cross the BBB using selectin and integrin ligands, while using cytokines and matrix metalloproteinases (MMPs) to further penetrate into the parenchyma^67,68^. There are few reports of CMV-induced changes to the BBB. Previously published data showed that mCMV infection leads to pericyte attraction, vessel maturation, and tightening of the BBB^23^. This process of partial restoration of the BBB might well physically exclude immune cells crossing into the tumor and thus contribute to immunosuppression and evasion^69^. As tolerance to the “missing-self” of NK cells is mediated by viral infection, TGF-β was reported to mediate blockade of TME infiltration and reduced NK proliferation through reduced activation^70^. The observed higher numbers of tumor infiltrating CD4 cells indicate their role in keeping tumor associated CMV reactivation in check. Similar observations have been made in patients with HIV/AIDS as antiretroviral therapy leads to the reestablishment of a competent immune system and anti-CMV therapy with ganciclovir can be discontinued as soon as the CD4 count is maintained above 100 cells/mm^3^^71^.

As the immunologic control breaks down, tumor cell proliferation overwhelms the immunological tumor control. This immune evasion ultimately leads to uncontrolled growth and impaired overall survival in the presence of CMV despite a competent immune system.

## Conclusions

mCMV exhibits only short periods of lytic replication in mouse glioblastoma cells. Changes to the transcriptome in these cells include the downregulation of MHC class I and II and a secretion of immunosuppressive cytokines such as IL-6, IL-8 and TGF-β. These changes are similar to those observed in CMV seropositive patients with glioblastoma. In vivo, mCMV is reactivated in orthotopic glioblastomas, leading to intratumoral viral spread, accelerated growth and shortened survival in a mCMV mouse model. The tumor immune landscape of mCMV + mice is dominated by B cells, with a lack of NK cells.

## Electronic supplementary material

Below is the link to the electronic supplementary material.

Supplementary Material 1

## Data Availability

All RNAseq data have been deposited in the European Nucleotide Archive (ENA) at EMBL-EBI under accession number PRJEB85259 (https://www.ebi.ac.uk/ena/browser/view/PRJEB85259).
