# Supplementary material for: Cytomegalovirus-induced oncomodulation drives immune escape in glioblastoma
Source: Sci Rep. 2025 Jul 17;15:25981. doi: 10.1038/s41598-025-10107-w (PMC12271317; doi:10.1038/s41598-025-10107-w)
Supplement: Supplementary file 1 — Supplementary Material 1 [file 41598_2025_10107_MOESM1_ESM.docx]

**Supplementary figure 1:** Cytokine expression after infection of mouse glioblastoma GL261Luc with mCMV leads to increased expression of predominantly IL-6 and IL-8.
